# Hepatoprotective effect of Zataria Multiflora Boisson cisplatin-induced oxidative stress in male rat

**Published:** 2015

**Authors:** A Ahmadipour, F Sharififar, F Nakhaipour, M Samanian, S Karami-Mohajeri

**Affiliations:** * Pharmaceutics Research Center, Kerman University of Medical Sciences, Kerman, Iran; ** Herbal and Traditional Medicines Research Center, Kerman University of Medical Sciences, Kerman, Iran

**Keywords:** Z. Multiflora, cisplatin, hepatoprotective, liver toxicity, antioxidant activity

## Abstract

**Background:** This research aimed to evaluate the protective effects of methanolic extract of Zataria Multiflora Boiss (Z. Multiflora) against hepatic damage induced by cisplatin in male Wistar rats.

**Methods:** Hepatotoxicity was induced in Wistar male rats by a single intraperitoneal administration of cisplatin, 7 g/ kg body weight. A methanolic extract of Z. Multiflora was administered orally at doses of 50 mg/ kg, 100 mg/ kg, 200 mg/ kg and 400 mg/ kg body weight daily for seven days after being cisplatin-induced. The study included the histopathological examination of the liver sections. The activity of aspartate transaminase (AST), alanine transaminase (ALT), and alkaline phosphatase (ALP) were evaluated as markers of liver damage. The superoxide dismutase (SOD), the activity of Catalase (CAT), and glutathione peroxidase (GSH-Px) and malondialdehyde (MDA) and nitric oxide (NO) content in serum were measured as an oxidative stress factor.

**Results:** The results showed that rat treated with cisplatin resulted in a significant increase in serum activity, AST, ALT and ALP in treated mice. Management with Z. Multiflora reduced the business of these enzymes to nearly normal levels. In parallel with these changes, this extract reduced cisplatin-induced oxidative stress by inhibiting lipid peroxidation and protein carbonylation, and restoring the antioxidant enzyme (SOD, CAT, and GSH-Px) and elevation of the glutathione level.

**Conclusion:** Biochemical and histological observations showed the hepatoprotective effect was found in a dose-dependent manner in Z. Multiflora methanolic extract. This protective effect can be attributed to the antioxidant compounds.

## Introduction

Cisplatin is one of the anticancer drugs that is frequently used for the treatment of various types of cancers [**1**,**2**]. However, dose-dependent hepatotoxicity induced by cisplatin limits the use of this useful drug [**3**-**6**]. Although renal cisplatin is recognized as the most important dose-limiting factor, little is known about liver damage caused by cisplatin [**7**]. The mechanism of alteration in liver function caused by cisplatin was not thoroughly revealed. Scientists believe that the oxidative damage to membrane lipids and other cellular components are an important factor in liver toxicity of cisplatin [**8**-**10**]. Cisplatin causes changes in the glutathione (GSH) status and increases lipid Peroxidation (LPO) [**11**-**14**]. The primary target for oxidative stress is induced by cisplatin mitochondria, resulting in the loss of mitochondrial protein SH, inhibiting the absorption of calcium, and decreasing mitochondrial membrane potential [**15**]. Hydrogen peroxide (H2O2), superoxide anion (O2•−) and hydroxyl radicals (OH•) are generated during oxidative stress [**16**,**17**]. The other radical producing mechanism is nitric oxide (NO) which reacts with O2•− to form peroxynitrite, a toxic agent to the cellular components [**18**]. Endogenous antioxidants that prevent the oxidative damage are involved in the superoxide dismutase (SOD), catalase (CAT) and glutathione peroxidase (GSH-PX) [**19**].

Medicinal plants are the primary source of a vast number of bioactive compounds used for the treatment of many diseases. Due to the presence of the antioxidant compounds such as phenols, flavonoids, tannins, many plants can be very useful in delaying or inhibiting the induction of oxidative stress [**20**-**22**]. The effect of these antioxidants as scavengers of free radicals was confirmed in several reports [**23**-**27**]. Zataria Multiflora Boiss (Z. Multiflora), belonging to the family Labiatae, is native in Iran and is used traditionally in food, especially in yogurt flavoring, as a stimulant, condiment, carminative [**28**]. Very high medicinal properties of this plant were reported, such as antioxidant, antimicrobial, analgesic, and anti-inflammatory activity [**29**]. The methanol extract and essential oil of Z. Multiflora have exhibited the considerable antioxidant effect that was attributed to phenolic compounds found in its extract [**29**]. This study investigated the protective effects of antioxidant chemistry Z. Multiflora against cisplatin-induced liver damage in male rats.

## Materials

In this research, adult male Wistar rats were used. Animals throughout the experiment were fed a standard commercial diet, kept at the room temperature, and held in a 12-hourlight/ 12-hour-dark cycle. All the analyses were performed by the “Guide for Care and Use of Laboratory Animals, DHEW Publication Done (NIH) 85-23, 1985”.

**Preparation of plant extracts**

Z. Multiflora plants were collected from the mountain of Shiraz city and were authenticated by Professor Fariba Sharififar of Kerman University of Medical Sciences, who has expertise in ethnobotany. Fresh aerial parts of the plants were washed under running water, shaded, and dried at room temperature (25 ± 2°C) for a week. Powder (250 g) was added in 750 ml of petroleum ether and then kept stirring for one night at room temperature. The supernatant extract was filtered, and the remaining plants were dried, the protocol being done with chloroform and then with methanol too. Finally, the methanol extract evaporated at 45°C with a rotary evaporator. The resulted dry powder was collected and stored in a refrigerator at 4°C for further use.

**Animal treatment **

For the experiment, the animals were classified into five groups (8 rats in each group as it follows: Normal control group received normal saline (10 ml/ kg body weight (b.w.)) by gavage for seven days. The active group received a single intraperitoneal (IP) dose of cisplatin of 7mg/ kg b.w. [**30**]. Three groups were treated with Z. Multiflora extract at different concentrations (50, 100, 200, and 400 mg/ kg b.w. by gavage) in normal saline (10 ml/ kg b.w.) for 7 consecutive days after a single IP dose of cisplatin. At the end of the therapy, all the rats were anesthetized with petroleum ether. Two ml of blood were taken through a cardiac puncture, and then serum was separated and rapidly frozen at −70°C for later analysis. Subsequent blood sampling, all the animals were killed, and the liver tissue was taken for biochemical analysis and histopathological examination. 

**Histopathological analysis**

A part of the right lobe of the liver was fixed in 10% neutral buffered formalin and processed for embedding in paraffin. Sections of 6µm thickness were cut and stained with hematoxylin and eosin for the evaluation of morphometric analysis and imaging by OLYMPUS photomicroscope.

**Serum aminotransferase levels**

To assess the damage of cell membrane of hepatocytes, the activity of the liver enzymes alanine transaminase markers (ALT) and aspartate transaminase (AST) were measured by the method described by Reitman and Frankel [**31**] and alkaline phosphatase (ALP) was based on the method presented by King and Armstrong [**32**].

**Lipid peroxidation assay**

The lipid peroxidation was estimated by using Beuge and Bust method [**33**] based on the measurement of total malondialdehyde (MDA) as the major product of lipid peroxidation. 

**Protein oxidation**

Protein oxidation was measured by estimating the total amount of the carbonyl group according to Levine and et al. method [**34**].

**Reduced glutathione level**

Glutathione (GSH) levels of liver were measured by using the modified method of Jollow Kalmanand et al. [**35**]. 0.5ml of 10% sulphosalicylic acid was added to a mixture of 0.4ml and 0.6ml of distilled water as homogeneous sediment protein. 0.5 ml of the supernatant of the reaction mixture, 4.5 ml and 0.5 ml of 0.5M Tris buffer of 10mm DTNB were mixed and the absorptions were read at 412 nm immediately. GSH content was calculated by using GSH as the standard and expressed as mmol/ of tissue.

**Superoxide dismutase (SOD) activity**

The total SOD activity was determined according to the method of Sun et al. [**36**]. It was based on the inhibition of nitro blue tetrazolium (NBT) reduction as a superoxide generator by xanthine-xanthine Oxidase system. The reagent contained 0.067 M Potassium phosphate buffer, pH 7.8, 0.1 M Ethylenediaminetetraacetic acid, and 1.5 mMNBT and the absorbances were read at 560nm. One unit of SOD was defined as the amount of enzyme that inhibited 50% of the reduction of NBT and was expressed asU/ mg protein.

**Catalase (CAT) activity**

The CAT activity was measured by the method of Johansson and et al. [**37**]. The reaction mixture contained 50mM phosphate buffer, pH 7.0, and three mM H2O2, and it was recorded spectrophotometrically at 240 nm. One unit of CAT was deﬁned as one nmol of degraded H2O2/ min/ milligram of protein.

**Nitric oxide determination**

Tissue nitrite (NO2-) and nitrate (NO3-) level were calculated as an indicator of nitric oxide (NO) by Griess reaction method [**38**]. Total nitrite (nitrite + nitrate) was measured spectrophotometrically at 545 nm after the conversion of nitrate to nitrite by copperized cadmium granules. A standard curve was established with a series of dilutions of sodium nitrite and results were reported as µmol/ gr of tissue.

**Protein measurement**

The protein concentration was evaluated according to the method of Bradford [**39**].

**Statistical analysis**

Data were analyzed by using the commercially available SPSS Software. Data were also analyzed by one-way ANOVA and Post hoc multiple comparison tests. Results were presented as means ± standard error (SEM). P-Values < 0.05 were regarded as statistically significant.

## Results and discussion

Histopathological finding

**Fig. 1:**
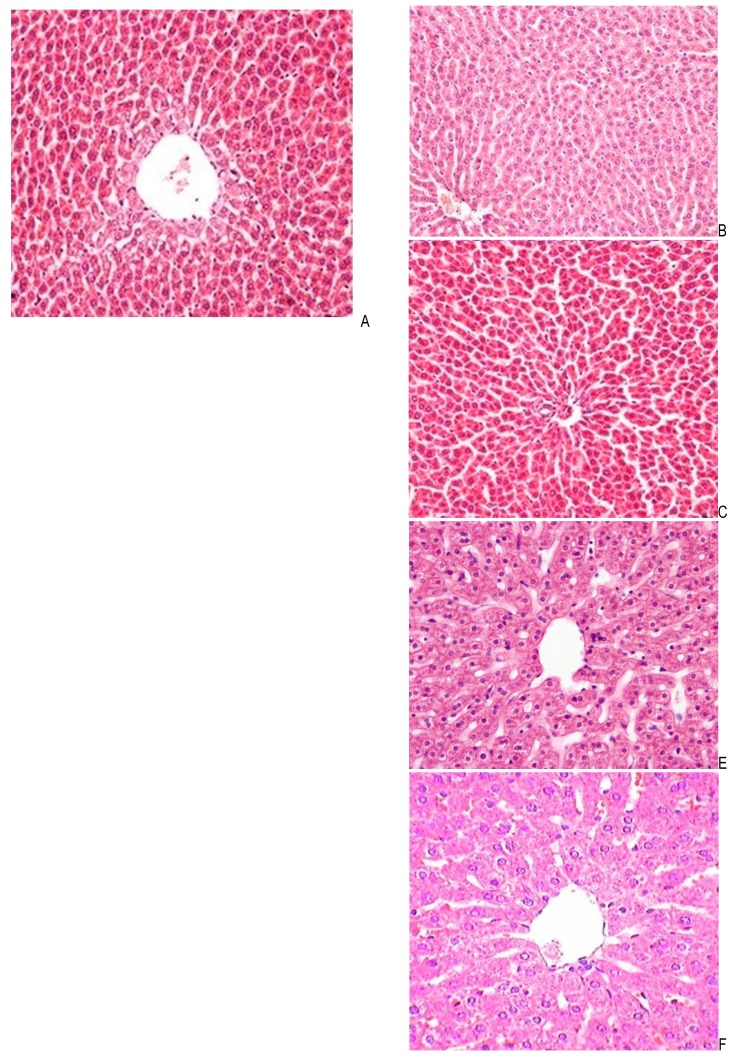
Photomicrographs of liver sections obtained from the normal group

**Fig. 2:**
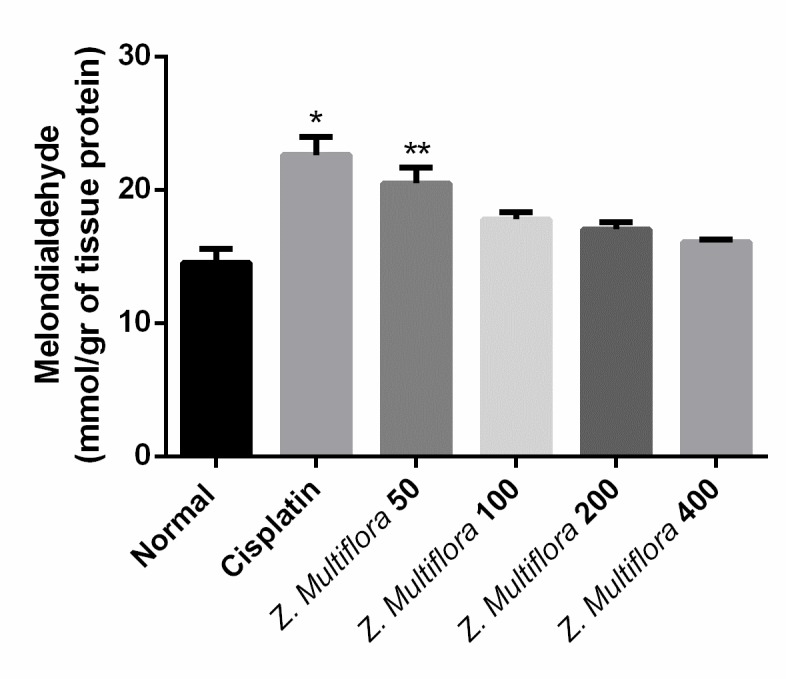
Cisplatin–induced group on the lipid peroxidation of liver tissue in comparison with positive and negative control groups

**Fig. 3:**
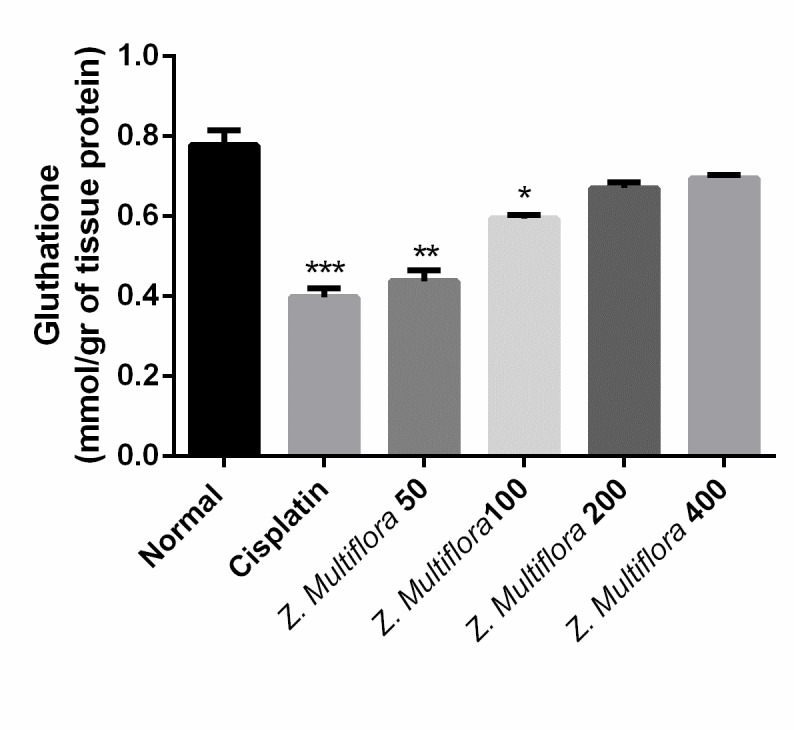
Effect of Cisplatin + Z. Multiflora (50 mg/ kg) group on the protein oxidation of liver tissue in comparison with positive and negative control groups

**Fig. 4:**
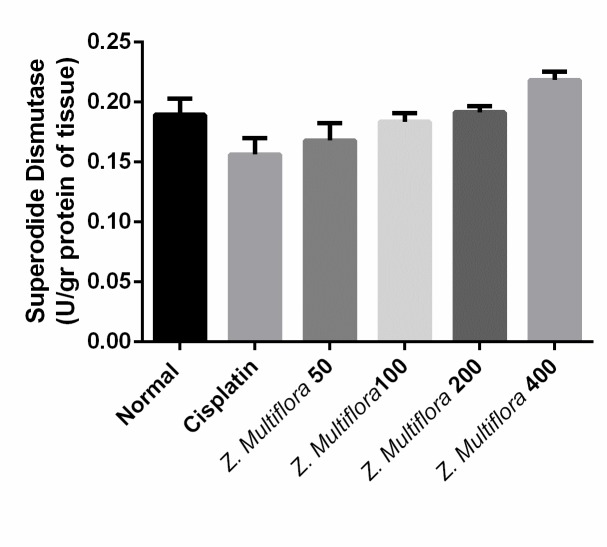
Effect of Cisplatin + Z. Multiflora (100 mg/ kg) on the glutathione content of liver tissue as compared to positive and negative control groups

**Fig. 5:**
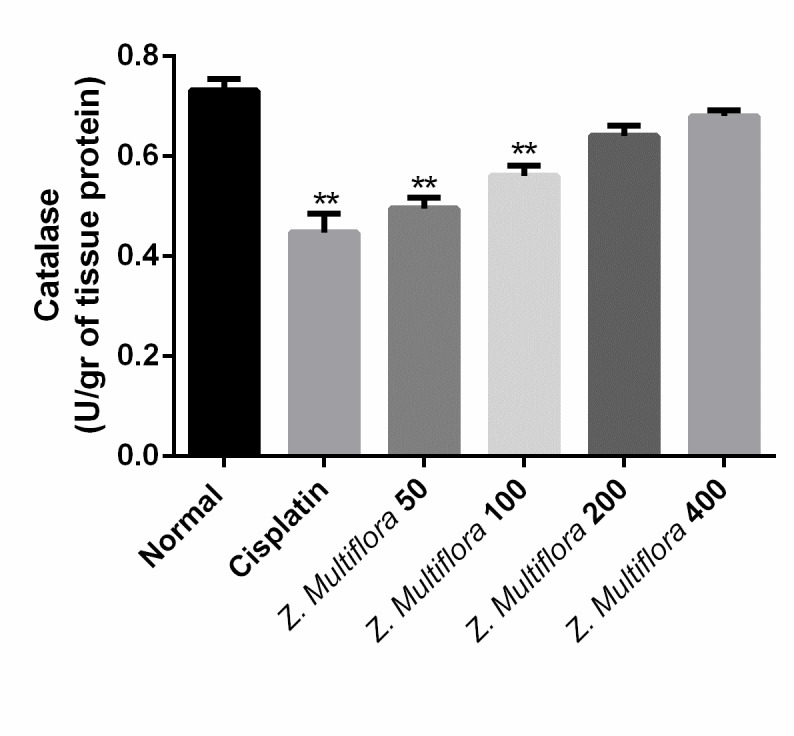
Effect of Cisplatin + Z. Multiflora (200 mg/ kg) group on the nitric oxide content of liver tissue in comparison with positive and negative control groups

**Fig. 6:**
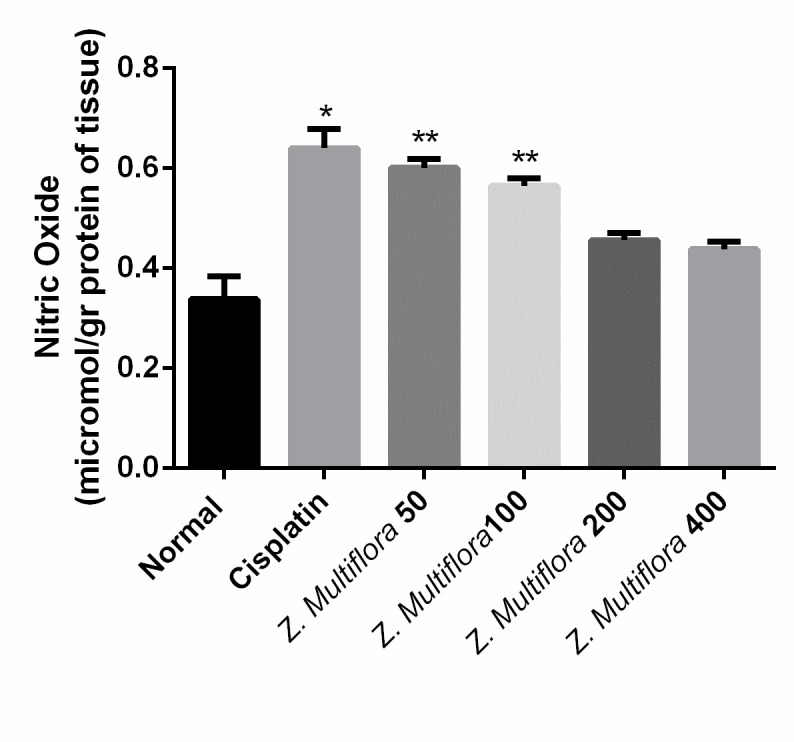
Effect of Cisplatin + Z. Multiflora (400 mg/ kg) group on the activity of superoxide dismutase enzyme in comparison with positive and negative control groups

The microscopic study of the liver tissue sections from the control group showed large polygonal cells with eosinophilic cytoplasm and round natural core arranged around the central vein and several hepatic sinuses (**Fig. 1**). In contrast, the second group received only cisplatin, which revealed obvious histological abnormalities including severe fatty change, central venous sinus congestion, and necrosis mainly around the central vein (**Fig. 2**). While the third and fourth group received 50 and 100 mg/ kg b.w. of Z. Multiflora methanolic extract along with cisplatin, it showed a regression of the fatty changes in the liver cell cytoplasm (**Fig. 3**,**4**). A greater improvement and the scattering of the apoptotic cells were seen at doses of 200 mg/ kgb.w. of Z. Multiflora methanolic extract (**Fig. 5**). The best results were observed in a dose of 400 mg/ kg b.w. of Z. Multiflora methanolic extract (**Fig. 6**).

**Serum aminotransferase levels **

The animals were treated with toxic doses of cisplatin that had markedly elevated values of the serum AST, ALT, and ALP compared to the normal group, indicating acute hepatocellular damage. The effects of the treatment with different doses of Z. Multiflora methanolic extract on serum AST, ALT, and ALP activities after the injection of cisplatin are shown in **Table 1**. The treatment with Z. Multiflora methanolic extract was continued for seven days after receiving the cisplatin reduced serum activities of AST, ALT, and ALP. The maximum decrease in serum AST, ALT, and ALP activities was observed in rats treated with 400 mg/ kg b.w. of Z. The Multiflora methanolic extract that significantly reduced serum AST, ALT, and ALP activities in the treated group were observed only in the cisplatin-induced group.

**Table 1:** Effect of various doses of Z. Multiflora on the activity of the serum aminotransferase enzymes in comparison with positive and negative control groups

	Normal	Cisplatin	Cisplatin + Zm 50 mg/ kg	Cisplatin + Zm 100 mg/ kg	Cisplatin + Zm 200 mg/ kg	Cisplatin + Zm 400 mg/ kg	Sig.
AST**(IU/ l)**	179.97 ± 24.61	414.36 ± 30.40	361.80 ± 37.13	284.08 ± 42.92	246.49 ± 37.49	238.96 ± 45.22	P <0.001
ALT**(IU/ l)**	59.59 ± 16.25	102.75 ± 16.25	93.48 ± 18.17	86.23 ± 28.08	71.23 ± 9.26	64.86 ± 11.33	P <0.001
ALP**(IU/ l)**	510.57 ± 39.44	803.38 ± 130.95	788.45 ± 94.92	709.33 ± 92.55	617.59 ± 40.58	582.67 ± 42.79	P <0.001

**Lipid peroxidation**

Tissue levels of MDA were significantly high in the cisplatin-treated group in comparison with the control group. On the other hand, Z. Multiflora reduced the level of MDA, and the best dose was 50mg/ kg. Results are shown in **Fig. 2**.

**GSH content**

GSH levels in the liver of the treated rats with only one injection of cisplatin showed a significant reduction (0.398 mg/ mg protein) compared to the standard group (0.778 mg/ mg protein). The treatment with methanol extracts of Z. Multiflora after the injection of a single dose of cisplatin significantly inhibited the reduction of the GSH level in the liver tissue, and the maximum effective dose was of 400 mg/ kg of methanol extracts.

**Antioxidant enzymes activities**

The activity of SOD and CAT in the liver of the cisplatin group was of 0.156U/ mg protein, 0.447 micromoles/min/mg protein and was better than the one in the cisplatin-induced group (0.189 U/ mg protein (P < 0.001), 0.731 micromoles/min/mg protein (P < 0.001). The results are shown in **Fig. 4** and **5**.

## Discussion

The hepatotoxicity of cisplatin is one of the major side effects of this drug [**40**]. Our histopathology findings in the liver tissue of the rat revealed cell degeneration, necrosis, and inflammatory infiltration on the seventh day after the execution of a single dose of cisplatin. Other findings in this study confirmed that the hepatocellular damage is the activity of serum aminotransferase, which was elevated in comparison with the control group. 

This study, like other studies, proved the induction of oxidative stress in liver tissue after cisplatin administration in rats [**7**,**12**,**41**-**44**]. The high dose of cisplatin caused a lipid peroxidation in liver tissue [**8**,**42**-**47**] and reduced the antioxidant enzymes activities, SOD, CAT, and GSH-PX, and GSH level of liver tissue. 

Reactive oxygen species and oxidative stress are thought to be a mechanism for cisplatin-induced cellular injury, so treatment and prevention strategies relying on antioxidants can be very helpful in the reduction of the hepatotoxicity side effect of cisplatin. Many studies have been done to establish a balance between oxidant and antioxidant system in cisplatin-treated cases by using a different group of antioxidants [**48**-**51**]. 

Along with other studies, this study also showed the hepatoprotective and antioxidant effects of methanolic extract of Z. Multiflora against cisplatin-induced liver damage in rats. Hepatocellular damage caused by cisplatin improved in groups received a metanolic extract of Z. Multiflora. The methanolic extracts of Z. Multiflora normalized the serum activities of ALT, ALP, AST, SOD, CAT, and GSH-Px, lipid peroxidation, and the GSH content in the liver.

Data from this study fully showed that the Z. Multiflora methanolic extract has a powerful effect on liver protection and the control of the toxicity increases in the serum, after the injection of Cisplatin in the rat in a dose-dependent manner. In addition, the use of this plant extract can be justified in the treatment of various diseases in traditional medicine because of its radical scavenging capacity. The drug profile of the extract on the clinical trials should be further investigated.

## Conclusion

Finally, based on this research, we can conclude that the Z. Multiflora methanolic extract causes a general protective effect against liver damage caused by cisplatin. Regarding the content of methionine and antioxidant properties, the protective effect of Z. Multiflora methanolic extract is regarded as a liberal radical scavenger, inhibitor of lipid peroxidation, and factor that decreases the glutathione levels.
